# Contaminating organic material in asbestos.

**DOI:** 10.1038/bjc.1969.46

**Published:** 1969-06

**Authors:** B. T. Commins, G. W. Gibbs


### CONTAMINATING ORGANIC MATERIAL IN ASBESTOS

B. T. COMMINS AND G. W. GIBBS

*From the Medical Research Council, Air Pollution Research Unit, St. Bartholomew's Hospital Medical College, London E.C.1 and the Department of Epidemiology and Health, McGill University, Montreal 2, Quebec*

Received for publication January 14, 1969

STUDIES have shown that several types of asbestos are carcinogenic; the subject has been reviewed (Gilson, 1966). The mechanism by which asbestos can induce cancer has not yet been established but it has been suggested that the small quantities of organic compounds commonly found in asbestos might be important (Harington and Roe, 1965; Harington, 1965). Tests using the organic material isolated from asbestos have shown that it is weakly carcinogenic to mice (Roe, Walters and Harington, 1966).

Natural and contaminating oils have been reported to be associated with all of the common forms of asbestos (Harington, 1962; Harington and Cilliers, 1963; Harington, 1965). It is known that contamination can occur in a number of ways, for example, when asbestos is stored in jute bags (Harington, 1965). We now report how asbestos can be contaminated by storage in polythene bags; this contamination comprises not only constituents of the polythene but also their oxidation products, produced when they make contact with the asbestos.

### EXPERIMENTAL

A sample of finely milled Canadian chrysotile was first examined; this had been used for biological studies in rats and had been stored in polythene. Using hot benzene, 71 mg. of a yellow oil was extracted from 350 g. of chrysotile. Spectroscopic examination after chromatographic separation showed the extract to contain traces of 3 : 4-benzopyrene, and, in the same chromatographic fraction, a yellow component with its main absorption maximum at 421 m$\mu$ and a lesser maximum at 400 m$\mu$. This yellow component was found in extracts of five other samples of asbestos (Union Internationale Contre Cancer (U.I.C.C.) reference samples (Report of a working group on asbestos and cancer, 1965) of crocidolite, amosite, anthophyllite, Rhodesian and Canadian chrysotile); the crocidolite and Canadian chrysotile samples contained the most and the anthophyllite the least. The chrysotiles yielded the most oil (up to 0·442 per cent w/w) and amosite the least (0·019 per cent w/w). Some additional samples of chrysotile fibre collected from the asbestos mills in Quebec and stored in polythene bags were also found to contain oil and the yellow component. In some other U.I.C.C. samples, less oil and much less of the yellow compound were found. All of these had been packed in polythene bags but those analysed earlier in the series had been stored in much smaller polythene bags than the later ones and we thought therefore that the yellow component might be related in some way to the polythene bags used for storing the samples. Some of the crocidolite and Rhodesian chrysotile samples originally stored in larger bags were packed into smaller bags made from the same type of polythene and after 1 week of storage a significant increase in the content of oil and yellow component was found. The yellow component could not be detected in extracts from unused polythene bags however and it seemed possible that it might be formed from some component of the adsorbed oil by chemical reaction on the surface of the asbestos.

Meanwhile, Dr. H. Powell and his colleagues at British Petroleum very kindly offered to examine by mass spectrum analysis the yellow component we had extracted from asbestos. They found it to contain compounds of mass numbers 408 and 410 having molecular formulae of $C_{28}H_{40}O_2$ and $C_{28}H_{42}O_2$. By comparing the mass spectrum they obtained with standard spectra they concluded that the compound having a mass number of 408 corresponded to 3,3′,5,5′ tetratertiary

(Reaction 1)

2,6-ditertiary butyl phenol (IV) — 3,3′,5,5′ tetratertiary butyl diphenoquinone (I)

butyl diphenoquinone (I), a yellow substance, which had ultraviolet and visible absorption spectra identical to those of the isolated yellow component. The other compound of mass number 410 corresponded to the colourless 4,4′-*bis*-(2,6-ditertiary butyl phenol) (II) which upon oxidation readily forms the diphenoquinone (I). To find out whether any constituent of polythene could be transformed into the quinone (I) we thoroughly extracted some finely milled crocidolite and after drying it added some cyclohexane washings of a polythene bag. In addition, portions of this asbestos were sealed in polythene bags. The results are shown in Table I. The results indicate that crocidolite asbestos can absorb oil

TABLE I.—*Oil and Tetratertiary Butyl Diphenoquinone Contents of Benzene Extracted Samples of Crocidolite After Special Treatment*

| Control | | Sample to which the cyclohexane washings of a polythene bag were added (after 1 week) | | Sample which was sealed in a polythene bag | | | |
|---|---|---|---|---|---|---|---|
| | | | | after 1 week | | after 6 weeks | |
| % oil | p.p.m. Q | % oil | p.p.m. Q | % oil | p.p.m. Q | % oil | p.p.m. Q |
| 0·003 | 0·01 | 0·226 | 0·60 | 0·037 | 0·34 | 0·058 | 0·66 |

[Q ≡ 3,3′,5,5′ tetratertiary butyl diphenoquinone]

from polythene and convert a constituent of the oil extracted from the polythene into diphenoquinone (I). Canadian chrysotile was found to act in a similar way to crocidolite.

Evidence that polythene bags were responsible for some but not all of the oils present in asbestos was obtained by collecting duplicate samples of Canadian chrysotile so that one sample could be stored in polythene and the other in a glass jar with an aluminium foil liner. The samples in glass jars gave consistently lower yields of oil than did those in polythene bags; the diphenoquinone (I) was detected in all of the polythene bag samples but in none of those kept in glass.

(Reaction 2)

2,6-ditertiary butyl paracresol (III) — 3,3′,5,5′ tetratertiary butyl stilbene quinone (V)

Polythene is usually made by polymerisation of ethylene dissolved in a paraffin of high molecular weight; anti-oxidants are commonly added. Fabricated polythene may contain some residues of paraffin and anti-oxidants which may be absorbed by asbestos. A common anti-oxidant used in the manufacture of polythene is 2,6-ditertiary butyl paracresol (III); it is of interest to note that 2,6-ditertiary butyl phenol (IV) can be readily oxidised (Hart, 1951) to the tertiary butyl diphenoquinone (I) which we have isolated from asbestos (reaction 1).

The phenol (IV) is unlikely to be present in commercial butyl cresol (III) commonly used in the manufacture of polythene. Oxidation of the butyl cresol (III) itself can however, under the right conditions (Cook, 1958), give rise to the diphenoquinone (I), but commonly some tetratertiary butyl stilbene quinone (V) is also produced (Kharasch and Hoshi, 1957); in cyclohexane this slightly higher

4,4′-*bis*(2,6-ditertiary butyl phenol) (II)

molecular weight quinone (V) has an adsorption peak at 449 m$\mu$ (reaction 2). Another phenol, 4,4′-*bis*(2,6-ditertiary butyl phenol) (II), can also be easily oxidised (Kharasch and Hoshi, 1957) to the diphenoquinone (I) and since this phenol has been identified by mass spectrum analysis in samples of oil extracted from asbestos it would seem possible that this is a precursor of the quinone (I).

2,6-ditertiary butyl para formyl phenol (VI)

para benzoquinone (VII)

 = tertiary butyl

Moreover, when either ditertiary butyl phenol (IV) or the corresponding cresol (III) are oxidised, a common intermediate, *bis* butyl phenol (II) may be produced. Another phenol, 2,6-ditertiary butyl para formyl phenol (VI) formed by oxidation (Cook, 1958) of the butyl cresol (III) can also be oxidised (Cook, 1958) to the diphenoquinone (I). The formyl phenol (VI) has been detected in aged polythene.

To investigate the possibility that asbestos could oxidise butylated phenols to diphenoquinone (I) small quantities (10–20 $\mu$g.) of the butyl phenol (IV) and the *bis* butyl phenol (II) were added to crocidolite and kept in stoppered glass jars; the asbestos was extracted with cold chloroform and it was found that over 50 per cent of both of these precursors of the diphenoquinone was oxidised within two hours. These results indicate that asbestos can promote the rapid oxidation of butyl phenol (IV) and *bis* butyl phenol (II) to diphenoquinone (I); either of these compounds might be present in polythene. *Bis* butyl phenol (II) is said to be used in the manufacture of some polyolefin plastics but not by the manufacturers of the polythene bags used in our studies. Tertiary butyl cresol (III) could be the immediate precursor of the diphenoquinone (I) but we have found that only traces of a mixture of the diphenoquinone (I) and the stilbene quinone (V) are formed in the presence of asbestos, and yet the stilbene derivative has not so far been detected in material extracted from asbestos. However, with one recently acquired sample of the butyl cresol (III) of uncertain purity a small proportion was converted to the diphenoquinone (I) only.

A possible immediate precursor of the quinone is butyl formyl phenol (VI) which has been detected in aged polythene and we have found that this substance can be slowly converted on crocidolite to the diphenoquinone (I). If the quantity of precursor is a function of the ageing of the polythene the amount of the diphenoquinone formed will increase with the time of storage of the asbestos in bags. The results shown in Table I suggest that this is so. The smallest amounts of the quinone (I) and oil would be expected to be found when large amounts of asbestos are stored for a short time in large bags (*i.e.*, where the ratio of the weight of asbestos to the surface area of the bag is large), this would account for the greater quantities of oil and quinone in the U.I.C.C. asbestos samples that had been packed in the smallest bags for periods of up to a year. The maximum concentration of the diphenoquinone (I) in any of the U.I.C.C. samples examined was 3·5 p.p.m. by weight in a sample of crocidolite.

### DISCUSSION

Although samples of asbestos which had been in contact with polythene have been shown to be carcinogenic to rats (Wagner, 1965) the biological significance of oil, of diphenoquinone (I) and of *bis* tertiary butyl phenol (II) found in asbestos stored in polythene has yet to be investigated. It is however worth pointing out that certain quinones, *e.g.*, para benzoquinone (VII) (a highly toxic compound [Patty, 1949]) when inhaled by rats is said to give rise to lung tumours (Hayashi, Kanisawa and Ide, 1963; Takizawa, 1940; Takizawa and Kanizawa, 1963; Kishzawa, 1954); the toxicity of quinones like the diphenoquinone (I) and the stilbene quinone (V) is low (I.C.I., 1968, private communication), but neither of these, nor the *bis* butyl phenol detected, have, so far as we know, been tested for carcinogenicity. Although the effects of individual additives to polythene and their oxidation products may be known, it is unlikely that they have been tested for biological action in the presence of asbestos fibre. At this stage we think it is reasonable to assume that any sample of asbestos in which the diphenoquinone is detected is contaminated, probably from storage at some time in polythene bags. Furthermore, it would seem unwise to use bags made of polythene to store materials intended for biological investigation or indeed for collection of geological specimens or meteorite fragments intended for subsequent organic analysis.

### SUMMARY

Evidence is presented that shows that asbestos contains organic material, some of which arises by contamination when the fibre has been stored in bags made of polythene. The contamination comprises not only the constituents of polythene, but also some of their oxidation products formed when they make contact with asbestos. Some of these products have been identified. It is known that organic material extracted from asbestos is weakly carcinogenic; it is not known however whether the oxidation products of some constituents of polythene are carcinogenic, but the implications of this finding are discussed.

We thank Professors P. J. Lawther and J. C. McDonald for advice and encouragement; Messrs. L. Hampton, H. Hui and F. Raveney for their skilled technical assistance; Dr. H. Powell and his colleagues at British Petroleum for their invaluable analytical assistance; the asbestos-producing companies of Quebec for help in many ways; and the Quebec Institute of Occupational and Environmental Health for financial assistance.

### REFERENCES

Cook, C. D.—(1958) *J. org. Chem.*, **23**, 755.
Gilson, J. C.—(1966) *Trans. Soc. occup. Med.*, **16**, 62.
Harington, J. S.—(1962) *Nature, Lond.*, **193**, 43.—(1965) *Ann. N.Y. Acad. Sci.*, **132**, 439.
Harington, J. S. and Cilliers, J. J. le R.—(1963) *Geochim. cosmochim. Acta.*, **27**, 411.
Harington, J. S. and Roe, F. J. C.—(1965) *Ann. N.Y. Acad. Sci.*, **132**, 439.
Hart, H.—(1951) *J. Am. chem. Soc.*, **73**, 3179.
Hayashi, Y., Kanisawa, N. and Ide, G.—(1963) *Jap. J. Cancer Clin.*, **2**, 167.
Kharasch, M. S. and Hoshi, B. S.—(1957) *J. org. Chem.*, **22**, 1439.
Kishzawa, F.—(1954) *Gann*, **45**, 389.
Patty, F. A.—(1949) 'Industrial hygiene and toxicology', Interscience.
Report of a working group on asbestos and cancer—(1965) *Br. J. ind. Med.*, **22**, 165.
Roe, F. J. C., Walters, M. A. and Harington, J. S.—(1966) *Int. J. Cancer*, **1**, 491.
Takizawa, N.—(1940) *Gann*, **34**, 158.
Takizawa, N., and Kanisawa, N.—(1963) *Jap. J. Cancer Clin.*, **2**, 172.
Wagner, J. C.—(1965) Proceedings of the third quadrennial international conference on cancer, University of Perugia.


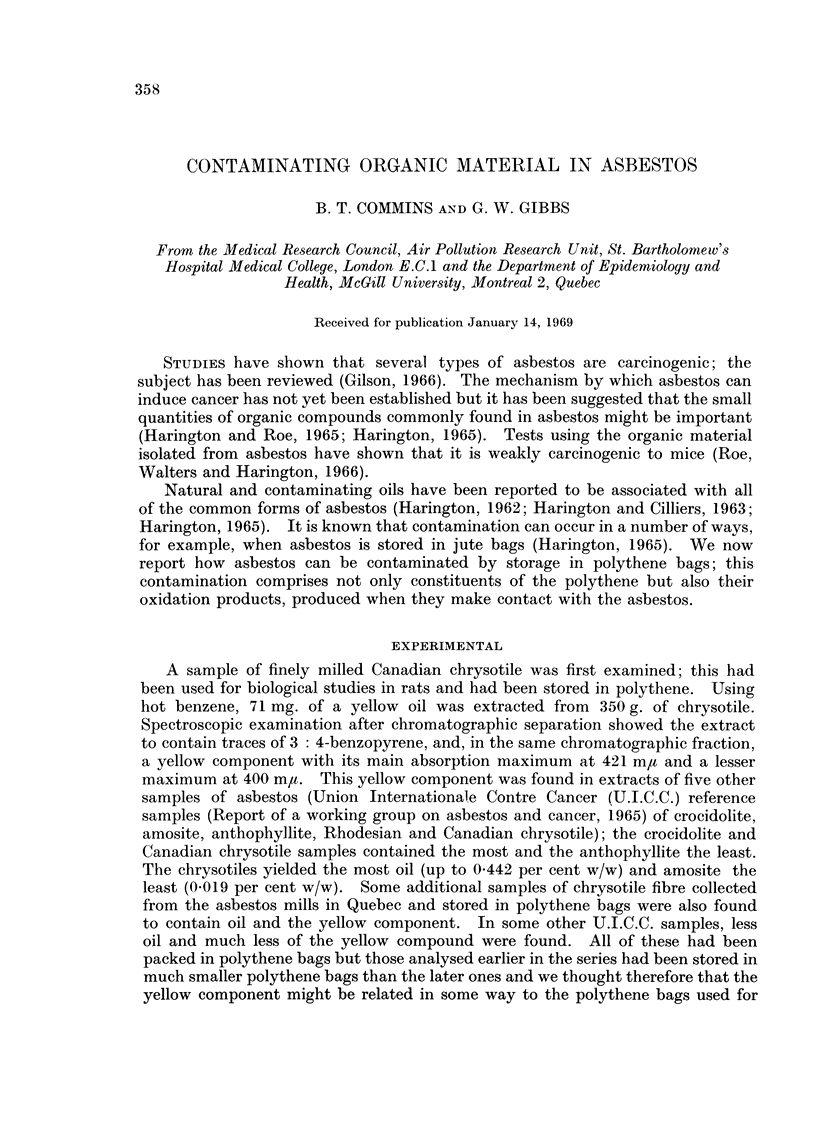

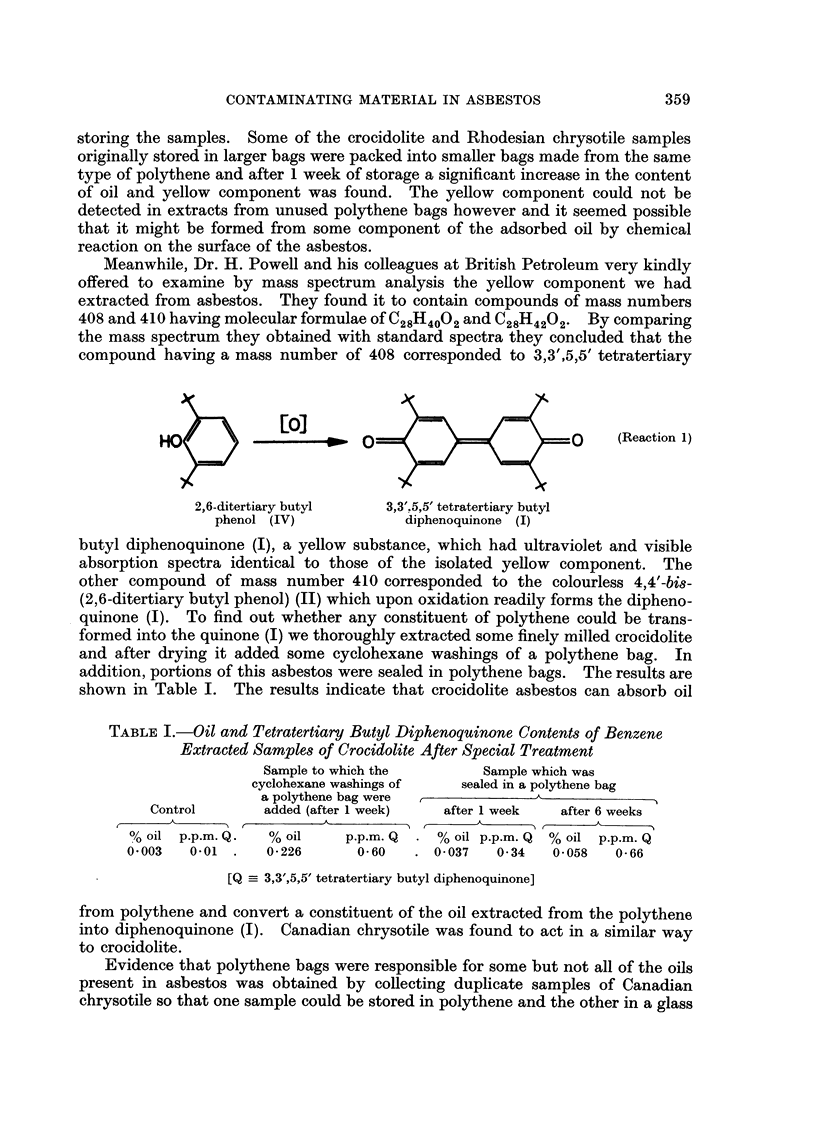

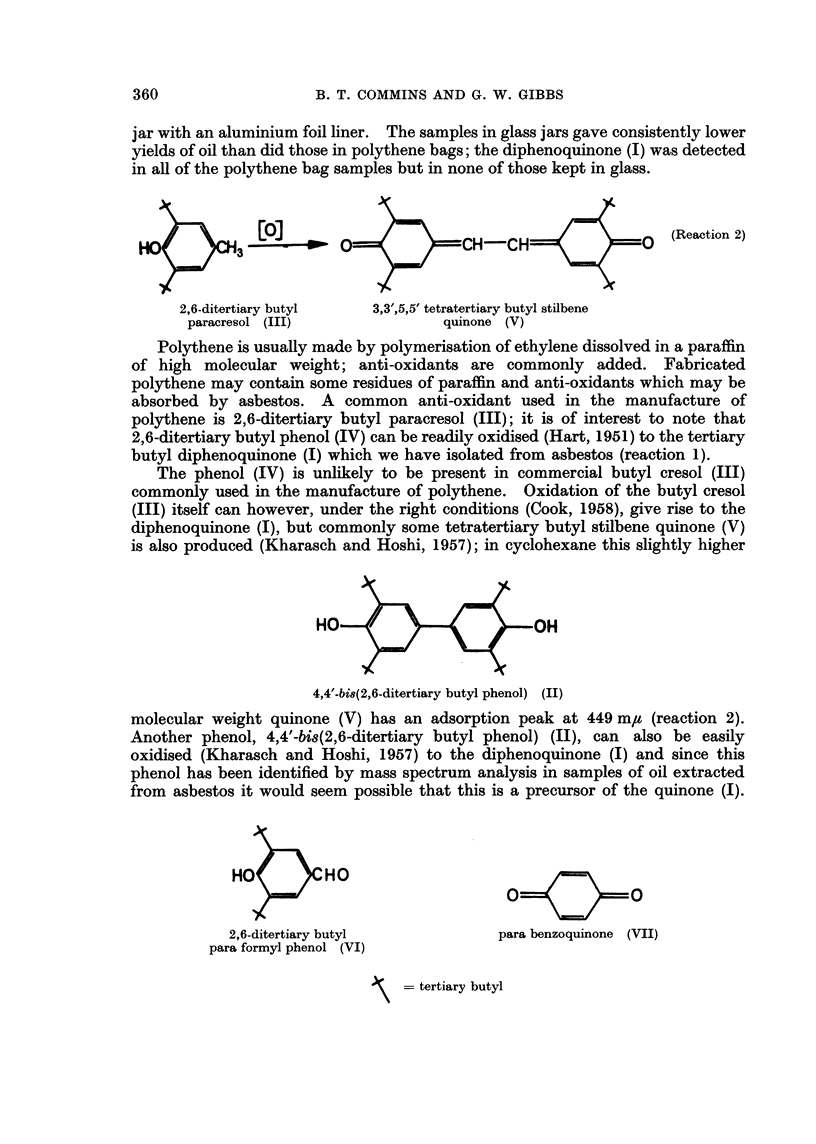

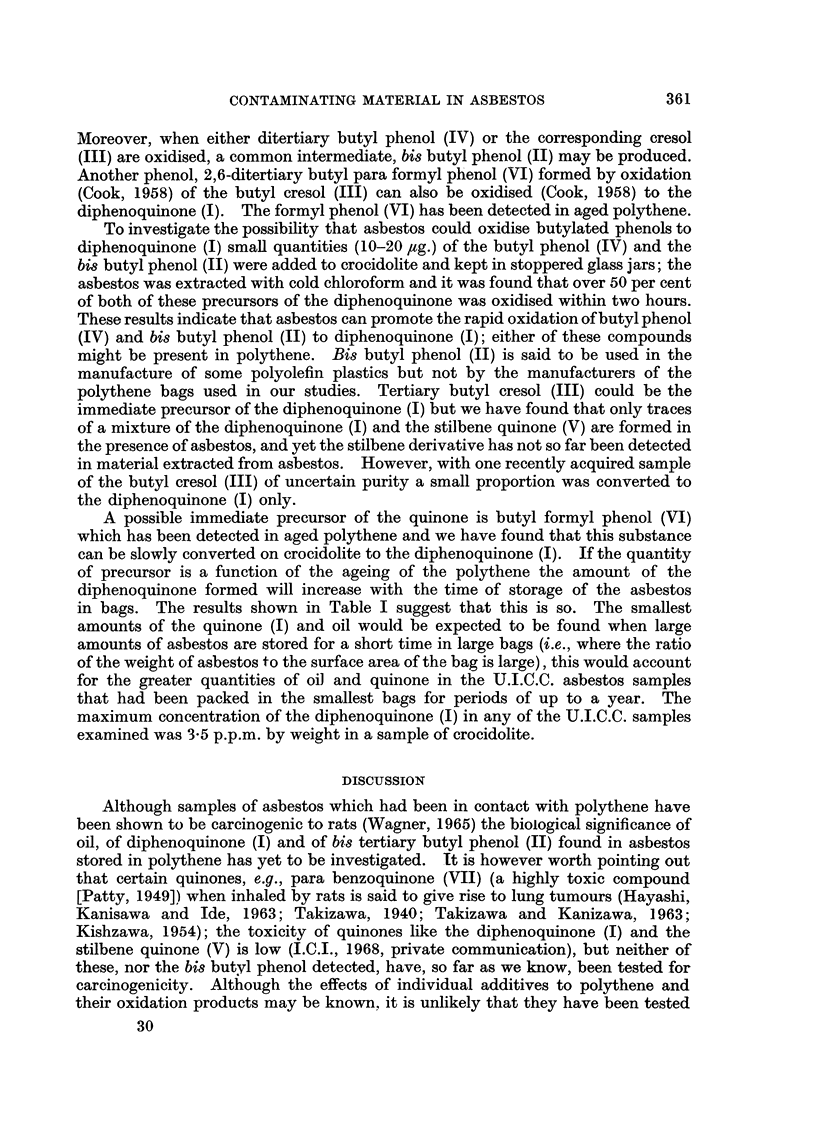

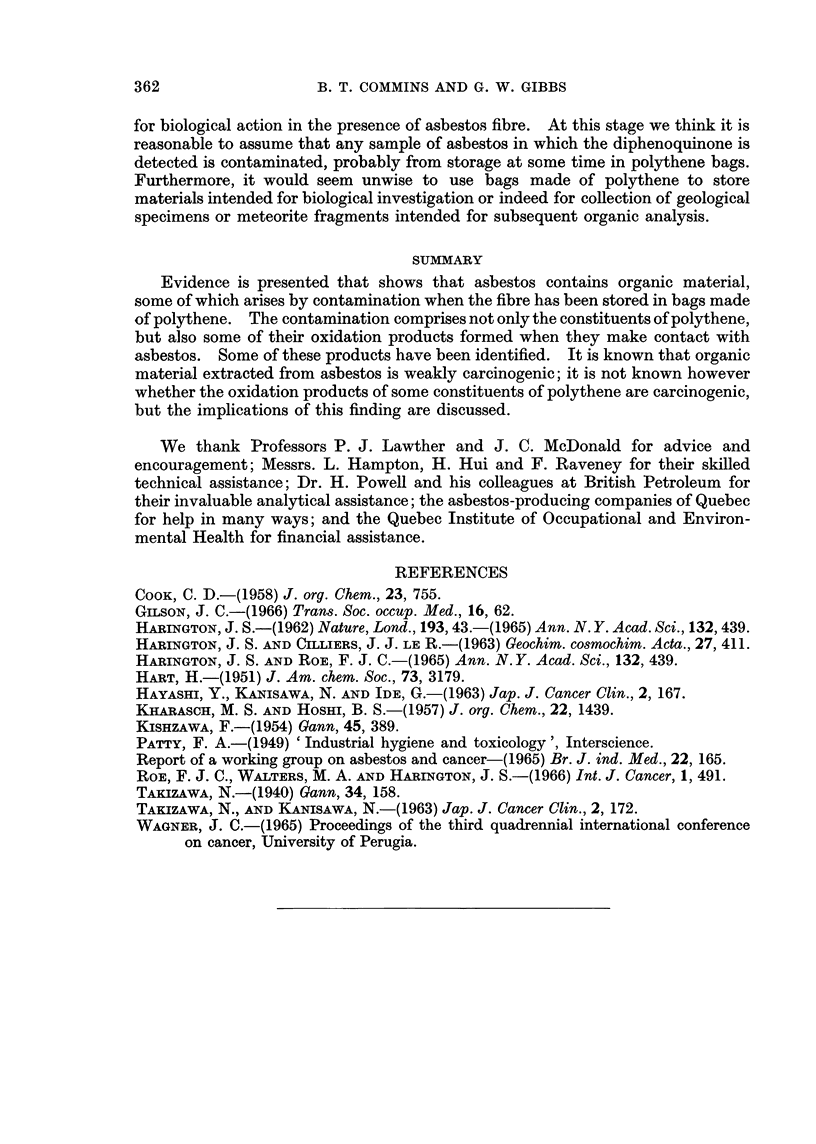


## References

[OCR_00249] Harington J. S., Roe F. J. (1965). Studies of carcinogenesis of asbestos fibers and their natural oils.. Ann N Y Acad Sci.

[OCR_00260] Roe F. J., Walters M. A., Harington J. S. (1966). Tumour initiation by natural and contaminating asbestos oils.. Int J Cancer.

